# Supplementary material for: Optimization of a natural fermentative medium for submerged mycelial culture of Ganoderma lucidum and the nutritional and bioactive composition of the cultured food-grade mycelia
Source: PLoS One. 2026 Jan 29;21(1):e0337539. doi: 10.1371/journal.pone.0337539 (PMC12854441; doi:10.1371/journal.pone.0337539)
Supplement: Attachment — Submitted filename: 20250627 PLOS ONE new version Ganoderma (1).doc [file pone.0337539.s002.doc]

**Optimization of a natural fermentative medium for submerged mycelial culture of *Ganoderma lucidum* and the nutritional and bioactive composition of the cultured food-grade mycelia**

**Shizhong Zheng^1, 2^, Lu Zhang^1, 4^, Yihui Yang^1, 4^, Shengrong Liu^1*^, Qi Wei^3^, Qianhui Huang^1^, Weirui Zhang^1,2^**

^1^ College of Biological Science and Engineering, Ningde Normal University, Ningde 352100, China

^2^ Fujian Higher Education Research Center for Local Biological Resources, Ningde 352100, China

^3^ College of Ocean, Ningde Normal University, Ningde 352100, China

^4^ College of Life Science, Fujian Agriculture and Forestry University, Fuzhou 350002, China

* Correspondence:

E-mail: fjhost@163.com

**Abstract**

*Ganoderma lucidum* is a famous traditional Chinese medicinal fungus with health-promoting benefits and pharmacological properties. To safely utilize the mycelia and broth of *G*. *lucidum* by submerged fermentation, a natural fermentative medium was formulated and optimized by the one-variable-at-a-time approach and response surface methodology (RSM). The nutritional and bioactive composition of the mycelia cultivated in the optimized medium and a nutrient-rich (NR) medium were compared. The one-variable-at-a-time experiments indicated that among various carbon sources, sucrose was favorable for mycelial growth. The additional supplementation of corn flour promoted mycelial growth and the optimal level was 7.0 g/L. A further optimization by RSM indicated that the optimal medium was comprised of 300 g/L potato (use its extract), 25 g/L sucrose, and 7.5 g/L corn flour. With the optimized medium, the mycelial yield reached 7.51 g/L. The crude ash (10.43%) and fiber (10.51%) content in the mycelia cultivated with the optimal medium was much higher than that (5.75% and 5.93%) of the mycelia grown in the NR medium, whereas the protein content and all examined amino acids were lower than those of the latter. The mycelia grown the optimized medium had 32.24 mg/g triterpenoids and 3.42% intracellular polysaccharides, significantly higher than that (20.35 mg/g and 2.03%) of the mycelia by growing in the NR medium. These findings proved that the food-grade cultured mycelia had the potential for use in the pharmacological industry because of its high content of triterpenoids and polysaccharides, and as a dietary supplement in the food and healthy products. The present study provides a reference for improving the economic benefits of *G*. *lucidum* submerged culture by using a natural medium, providing the condition for the safe utilization of all fermentation products, and thus its large-scale industrial fermentation may be economically feasible.

**Introduction**

*Ganoderma lucidum* (Fr.) Karst belonging to the family Polyporaceae, commonly known as Lingzhi in Chinese, is a medicinal fungus that is widely used to prevent and treat various diseases such as asthma, bronchitis, hepatitis, hypertension, hepatopathy, arthritis, nephritis, gastric ulcer and cancers in oriental countries for centuries [1]. *G*. *lucidum* possesses diverse biological and pharmacological activities, including antitumor, hypotensive, hypoglycemic, immune modulatory, anti-inflammatory, skin lightening, neuroprotective, and anti-oxidative effects [1, 2]. In the fruit bodies, spores, and mycelia of *G*. *lucidum,* a wide range of physiologically active substances have been isolated. These include polysaccharides, triterpenoids, steroids, alkaloids, and nucleotides [3, 4]. Among these compounds, the polysaccharides and triterpenoids are the two most important bioactive constituents.

To meet the high demand for high-quality *G*. *lucidum* in market, its artificial cultivation on short trunks or synthetic substrates is a widely adopted method for the production of fruiting bodies. With the fruiting bodies as raw materials, a variety of *G*. *lucidum* products such as slices, powder, tablets, capsule, and injections could be manufactured [5]. Presently, the artificial cultivation of *G*. *lucidum* is prosperous in China, Korea, India and some Southeast Asian countries. However, there are several disadvantages associated with solid cultivation, including long cropping cycle, unstable product quality, requirement of a large space, and a high influence of environmental changes on the cultivation [6, 7].

Submerged culture of mushrooms has the potential of high biomass yield in a compact space over a short time with less risk of contamination [8]. Hence, it has been viewed as a promising alternate to solid cultures for the production of mushrooms. In submerged fermentation of mushrooms, the mycelia and small quantities of several useful metabolites could be obtained. As *G*. *lucidum* has perceived healthy-promoting effects and high medicinal value, considerable attention has been paid to its submerged cultures for the production of mycelia, polysaccharides and triterpenoids. To improve the productivity, several strategies such as optimization of medium and culture conditions [9, 10], development of novel processes [11, 12], elicitation by addition of inducers [13], genetic manipulation of strains [14, 15], and development of solid seed [16] have been explored, and some improvements have been achieved. However, from a viewpoint of industrial scale application, the productivity of mycelia and bioactive metabolites are still low in submerged fermentation of *G*. *lucidum*, making the process industrially unattractive. Increasing product yields and developing novel production systems by solving the existing problems associated with submerged fermentation technique of mushrooms are considered to be crucial for its success on a commercial scale [17]. In submerged fermentation of mushrooms, utilizing all the fermentation products including mycelia and culture broth may be another solution to improve bioprocess economy, but the effect of medium compositions on the safety of fermentation products has not yet been considered when they were selected.

The objective of the present study was to develop a natural medium for submerged mycelial culture of *G*. *lucidum* from a perspective for the safe utilization of fermentation products (mycelia and culture broth). For this purpose, a food-grade medium was typically formulated using agricultural products and food ingredients, and optimized using the empirical one-variable-at-a-time approach and response surface methodology. In order to provide a scientific basis for utilizing the cultured food-grade mycelia harvested from the developed natural medium, the content of nutritional and bioactive composition in the mycelia was investigated. The present study provides a new perspective for improving the economic benefits of *G*. *lucidum* submerged fermentation by using a natural medium, ultimately leading to the safe utilization of all fermentation products, and thus its large-scale industrial fermentation may be feasible.

**Materials and methods**

**Mushroom strain and medium**

*Ganoderma lucidum* G10016, obtained from the Mycological Research Centre of Fujian Agriculture and Forestry University (Fuzhou, China), was used in this study. It was incubated at 25°C for 7 days on potato dextrose agar (PDA) slants, stored at 4°C, and sub-cultured every four weeks.

**Media**

PDA medium comprised of 200 g potato, 20 g glucose, and 15-18 g agar in 1 L distilled water. Potato dextrose broth (PDB) was PDA medium by omission of agar. Nutrient-rich (NR) medium was comprised of 30 g glucose, 5 g peptone, 4 g yeast extract, 1 g KH_2_PO_4_, and 0.5 g MgSO_4_ in 1 L distilled water.

**Inoculum preparation**

Ten mycelial agar discs (7 mm in diameter), cut from the colony edge of the growing cultures in PDA plates, were inoculated into a 250 mL Erlenmeyer flask with 50 mL of PDB medium, and shaken in a rotary incubator at 150 rpm and 25°C for 5 days. Then, the mycelial culture was homogenized with an ultrasonic cell disrupting apparatus (SCIENTZ-IID, Ningbo Scientz Biotechnology, Co., Ltd., Ningbo, China) with power input of 550 W and frequency of 20 kHz using a 6 mm diameter horn-type probe. The homogenate (5 mL) was transferred to 50 mL PDB in a 250 mL flask, and cultivated further for 2 days. The finally obtained mycelial culture was used as liquid seed in subsequent studies.

**Submerged culture**

All submerged cultures in this study were performed in 250 mL Erlenmeyer flasks with 80 mL medium. The inoculation of liquid seed was set at 10% (v/v). The flasks were cultivated at 25°C and 150 rpm for 4 days. The samples were taken for harvesting mycelia.

**One-variable-at-one-time experiments**

PDB medium was used as a basic medium. To select a suitable carbon source for mycelial yield of *G*. *lucidum* in submerged cultures, the glucose in the medium was replaced individually with sucrose, fructose, starch, and maltose, each at 20 g/L. After this, the effect of a low-cost ingredient corn flour on the mycelial growth was tested in the range of 1.0 to 11.0 g/L with the optimal carbon source in the medium, and the optimal level was determined.

**Response surface experimental design**

According to the results of one-variable-at-a-time experiment, the medium compositions, i.e., potato, sucrose, and corn flour were considered as independent variables, and their levels were optimized for maximizing mycelial production by response surface methodology (RSM) with Box-Behnken design (BBD). Design-Expert version 8.0 was used for experiment design, data analysis, mapping, and optimization.

The symbols, the actual and coded values of the independent variables are shown in Table 1. Each variable was tested at three levels, coded as low (-1), medium (0), and high (+1) levels, respectively.

**Table 1. Range and levels of the variables investigated in the BBD for biomass production of *G*. *lucidum.***

| Variables | Symbol | | Range and levels | | |
| --- | --- | --- | --- | --- | --- |
|  | Uncoded | Coded | -1 | 0 | 1 |
| Potato (g/L) | *x_1_* | *X_1_* | 100 | 200 | 300 |
| Sucrose (g/L) | *x_2_* | *X_2_* | 15 | 25 | 35 |
| Corn powder (g/L) | *x_3_* | *X_3_* | 2.5 | 5 | 7.5 |

*X_1_*= (*x_1_*-100)/100,  *X_2_*= (*x_2_*-25)/10, and *X_3_*= (*x_3_*-5)/2.

The matrix of BBD design is shown in Table 2. The design consisted of a total of 17 runs, with three replicates at the center point to estimate the pure error of the experiments. The biomass yield was taken as the response variable. To correlate the response variable to the independent variables, the results were fitted into a following second-order polynomial equation using a multiple regression technique.

Y = *β*_0_ + $\sum_{i=1}^{n} \beta_{i}X_{i}$+ $\sum_{i=1}^{n} \beta_{\text{ii}}X_{i}$+$j>i\underset{n-1}{}X_{j}$+ ε

where *Y* is the predicted biomass yield, *β*_0_ is the model intercept term, and *β_j_*, *β_jj_* and *β_ij_* are the regression coefficients for linear, quadratic, and interaction terms, respectively. *X_i_* and *X_j_* are the coded variables. ε represents the error for the system.

**RSM modeling and statistical checking**

The statistical significance of the equation was determined by an *F*-test, and the analysis of variance (ANOVA) for response surface quadratic model was performed to test the significance of each coefficient in the equation. The fitness of the model was evaluated by the determination coefficient *R^2^*, adjusted determination coefficient (Adj *R^2^*), and the Lack of Fit *P*-value. The *P* value was used as a tool to check the significance of each of the coefficients and indicates the interaction strength between the independent variables. Values of *P* less than 0.05 were statistically significant. Three-dimensional response surfaces and two-dimensional contour plots of the regression equation were drawn to illustrate the relationship between the response and experimental levels of each independent variable and the type of interactions between the variables.

**Validation of mathematical model**

To evaluate the fitted model, validation experiments were carried out with the optimized medium. The difference in biomass yield between the experimentally obtained and predicted values was used to evaluate the accuracy and general availability of the model.

**Fermentative production of mycelia**

For production of mycelia, submerged fermentation was carried out in flasks with the optimized medium. Submerged cultivation was also performed using the NR medium. The mycelial cultures were filtered for harvesting mycelia, and washed with distilled water. The clean mycelia were lyophilized, and ground into a fine powder. The mycelial powder were used for analysis of the nutritional and bioactive compositions.

**Measurement of biomass**

To ensure the accurate measurement of mycelial biomass, three independent culture flasks were taken as replicates. All the mycelia in each culture flask was collected by filtration on gauze, followed by repeated washing with distilled water, and drying at 60°C to a constant weight. The dry mycelial weight was measured on an electronic balance.

**Proximate composition analysis**

The proximate composition of the cultured mycelia of *G*. *lucidum*, including moisture, crude ash, crude fiber, crude fat, and crude protein, were determined according to methods described by Ouzouni et al. [18]. Ash was determined by combustion in a muffle furnace at 600°C. The protein content was determined with the micro Kjeldahl method, and the nitrogen factor used for crude protein calculation was 4.38. The crude fat content was determined by extraction with hexane using a Soxhlet system. The total carbohydrate content is calculated by subtracting the contents of ash, fat, and protein from 100. Amino acid analysis was carried out after hydrolysis of the mycelia with 6 mol/L HCl for 24 h at 110°C using an amino acid analyzer.

**Determination of polysaccharides**

To estimate the intracellular polysaccharides, the phenol-sulfuric acid method was used [19]. The powdered mycelia (100 mg) was extracted in 5 mL 1 mol/L NaOH at 60°C for 1 h, and then centrifuged at 8000 ×g for 10 min. The resulting supernatant was used in the reaction. A standard curve was built using D-glucose as the standard and the content of intracellular polysaccharides in the mycelia was calculated.

**Determination of triterpenoids**

For the quantification of total triterpenoids, the vanillin-glacial acetic acid method described by Cai et al. [20] was used. The dried mycelia were ground in a mortar with pestle and sieved with a 60-mesh stainless screen to obtain a fine powder. The powder (100 mg) was extracted twice in 5 mL 80% ethanol in one week. The extracts were combined and centrifuged, and the resulting supernatants were used for analysis. To calculate the triterpenoids, a standard curve was built with ursobic acid as a standard.

**Statistical analysis**

All the experiments were carried out in triplicates, and the results were expressed as the mean ± standard deviation. Significant differences were determined by Duncan’s multiple range tests (*P* < 0.05).

**Results**

**Preliminary optimization of medium compositions by one-variable-at-a-time approach**

**Screening of a suitable carbon source for biomass production**

Fig 1 shows the biomass production of *G*. *lucidum* in response to different carbon sources in submerged culture. Among the tested carbon sources, sucrose gave the highest biomass yield of 3.44 g/L, and maltose, glucose and fructose supported moderate mycelial growth, and the least growth of only 1.68 g/L was observed with starch. Considering the biomass yield, material cost as well as availability, sucrose was therefore selected as a favorable carbon source for biomass production in subsequent experiments.

**Fig 1. Effects of different carbon sources on the biomass production in *G*. *lucidum*** **submerged cultures.** The different letters on the error bars represent significant differences (*P* < 0.05).

**Effects of corn flour supplementation on biomass production and its optimal level**

With sucrose instead of glucose as carbon source in PDB medium, the effect of supplementation of inexpensive, easily available corn flour on the mycelial growth of *G*. *lucidum* was evaluated. Fig 2 shows that the additional supplementation of corn flour in the medium greatly enhanced biomass production, and the enhancement effect was closely related to the supplemented level. The maximal biomass production reached 5.21 g/L when corn flour was supplemented at 7.0 g/L, 52.61% higher than that obtained in the medium (3.44 g/L) without addition. Hence, corn flour is a suitable nutrient composition for promoting biomass production in submerged fermentation of *G*. *lucidum*.

**Fig 2. Effects of corn flour supplementation on the biomass production in *G*. *lucidum* submerged cultures and its optimal level.** The different letters on the error bars represent significant differences (*P* < 0.05).

**RSM model fitting of biomass yield and statistical significance**

Potato, sucrose, and corn flour were selected as independent variables in BBD design for further optimization by RSM. The design matrix of BBD along with the corresponding response values of biomass yield were shown in Table 2. The biomass production varied significantly among 17 runs, with the minimum biomass of 3.76 g/L on Run 1, and a maximum value of 7.26 g/L by Run 5. The difference exhibited that the three studied variables and their interactions had a great impact on biomass production of *G*. *lucidum* in submerged culture.

**Table 2. The matrix of Box-Behnken design with coded and actual (in parentheses) values of the three independent variables along with experimental results of biomass production**.

| Run | Independent variables | | | Biomass (g/L) |
| --- | --- | --- | --- | --- |
|  | *X_1_* (potato) | *X_2_* (sucrose) | *X_3_* (corn flour) |  |
| 1 | –1 (100) | 0 (25) | –1 (2.5) | 3.76 ± 0.13 |
| 2 | 0 (200) | 0 (25) | 0 (5.0) | 5.86 ± 0.15 |
| 3 | 0 (200) | 1 (35) | –1 (2.5) | 4.06 ± 0.21 |
| 4 | 0 (300) | –1 (15) | 0 (5.0) | 5.04 ± 0.09 |
| 5 | –1 (100) | 1 (35) | 0 (5.0) | 4.50 ± 0.17 |
| 6 | 1 (300) | 0 (25) | 1 (7.5) | 7.26 ± 0.24 |
| 7 | 0 (200) | –1 (15) | 1 (7.5) | 6.44 ± 0.14 |
| 8 | 0 (200) | 0 (25) | 0 (5.0) | 6.10 ± 0.22 |
| 9 | 0 (200) | 0 (25) | 0 (5.0) | 6.22 ± 0.18 |
| 10 | 0 (200) | 1 (35) | 1 (7.5) | 6.50 ± 0.14 |
| 11 | 1 (300) | 1 (35) | 0 (5.0) | 7.04 ± 0.18 |
| 12 | –1 (100) | 0 (25) | 1 (7.5) | 5.48 ± 0.15 |
| 13 | 0 (200) | 0 (25) | 0 (5.0) | 6.24 ± 0.11 |
| 14 | –1 (100) | –1 (15) | 0 (5.0) | 4.50 ± 0.21 |
| 15 | 0 (200) | 0 (25) | 0 (5.0) | 5.76 ± 0.26 |
| 16 | 1 (300) | 0 (25) | –1 (2.5) | 4.74 ± 0.17 |
| 17 | 0 (200) | –1 (15) | –1 (2.5) | 4.08 ± 0.20 |

Thought multiple regression analysis on the experimental data, a fitted second-order quadratic polynomial equation with three variables in coded values was established as follows:

*Y* = 6.04+0.73*X_1_*+0.26*X_2_* +1.13*X_3_*+0.50*X_1_X_2_*+0.20*X_1_X_3_*+0.02*X_2_X_3_*–0.36$X_{1}^{2}$–0.40$X_{2}^{2}$– 0.36$X_{3}^{2}$

The ANOVA results of the fitted quadratic equation are shown in Table 3. The quadratic regression model was highly significant, as is evident from a high model *F*-value of 19.65 and a very low probability value (*P*<0.0004). The Lack of Fit *F*-value of 3.8 was low and its *P*-value (0.1149) was greater than 0.05, implying that the lack of fit was statistically insignificant, and the model is not related to the pure error. The determination coefficient *R^2^* was high, reaching 0.9619, indicating that 96.19% of total variation of the biomass yield could be explained by the model. The adjusted *R^2^* (Adj *R^2^*) was in reasonable agreement with *R^2^*, indicating a high correlation between the experimental and predicted values. The linear effect of potato (*X_1_*) and corn flour (*X_3_*) and the interactions of *X_1_×X_2_* were significant. All quadratic terms (*X_1_^2^*, *X_2_^2^*, and *X_3_^2^*) had no significant effect on biomass production. The coefficient of variation (CV) of 5.82% was low, indicating the accuracy and reliability of the experiments performed.

**Table 3. ANOVA results of the fitted quadratic model for the biomass yield in *G*. *lucidum* submerged cultures.**

| Source | Sum of squares | DF | Mean square | *F*-value | *Prob*>*F* |
| --- | --- | --- | --- | --- | --- |
| Model | 18.16 | 9 | 2.02 | 19.65 | 0.0004 |
| *X_1_*: Potato | 4.26 | 1 | 4.26 | 41.51 | 0.0004 |
| *X_2_*: Sucrose | 0.52 | 1 | 0.52 | 5.07 | 0.0591 |
| *X_3_*: Corn powder | 10.22 | 1 | 10.22 | 99.46 | <0.0001 |
| *X_1_* × *X_2_* | 1.00 | 1 | 1.00 | 9.74 | 0.0168 |
| *X_1_* × *X_3_* | 0.16 | 1 | 0.16 | 1.56 | 0.2521 |
| *X_2_* × *X_3_* | 1.600E-003 | 1 | 1.600E-003 | 0.016 | 0.9042 |
| *X_1_^2^* | 0.55 | 1 | 0.55 | 5.40 | 0.0531 |
| *X_2_^2^* | 0.68 | 1 | 0.68 | 6.66 | 0.0365 |
| *X_3_^2^* | 0.55 | 1 | 0.55 | 5.40 | 0.0531 |
| Residual | 0.72 | 7 | 0.10 |  |  |
| Lack of fit | 0.53 | 3 | 0.18 | 3.80 | 0.1149 |
| Pure error | 0.19 | 4 | 0.047 |  |  |
| Cor total | 18.88 | 16 |  |  |  |
| *R^2^* | 0.9619 |  |  |  |  |
| Adj *R^2^* | 0.9130 |  |  |  |  |
| Pred *R^2^* | 0.5336 |  |  |  |  |
| Adeq precision | 15.135 |  |  |  |  |
| CV | 5.82% |  |  |  |  |

**Response surface analysis of the independent variables on biomass production**

The graphical representations of the fitted model, called response surface curve and contour plots, are illustrated in Fig 3. These graphs demonstrated the individual and interactive effects of the three investigated compositions on biomass production of *G*. *lucidum* in submerged cultures. The three-dimensional response surface plots (Fig 3A) indicated that high levels of both potato (*X_1_*) and sucrose (*X_2_*) were favorable for mycelial growth, but the two variables’ degree of influence was not high as the slope of the curvature was relatively flat. An elliptical nature of contour plots between potato and sucrose indicated that the interactive effect of these two variables on biomass production was significant.

Fig 3B shows the combined effects of potato (*X_1_*) and corn flour (*X_3_*) on biomass production while keeping the third variable (*X_2_*, sucrose) at zero level. The response surface curves display that increasing amount of either potato or corn flour inside the limits of the investigated region can increase biomass yield. The interaction between potato and corn flour (*X_1_X_2_*) was negligible, as the shape of contour plots was circular in nature (Fig 3b).

The curvature of response surfaces in Fig 3C is relatively flat, and its corresponding contour plots are circular in shape (Fig 3c). The response surface and contour plots indicated that there were no significant interactions between sucrose and corn flour. Changing sucrose concentration within the investigated range had little effect on biomass yield. whereas a small variation of corn flour concentration could lead to a remarkable impact on the response variable, with the preference at higher concentration. A high biomass production could be obtained at a relatively high level of corn flour (7.5 g/L) when keeping sucrose at the vicinity of its zero level (25.0 g/L).

With the numerical optimization technique of the software Design-Expert and based on response surface analysis, the optimal concentration of the three compositions for maximal biomass yield was attained as 300 g/L potato, 25 g/L sucrose, and 7.5 g/L corn flour.

**Fig 3. Response surface (A, B, and C) and contour (a, b, and c) plots showing the individual and interactive effects of the three medium components on the biomass production of *G*. *lucidum* in submerged cultures.**

**Validation of the mathematical model**

To validate the predictive model, verification experiments were carried out using the optimized medium. The biomass production reached 7.51 g/L, close to the predicted value of 7.37 g/L, implying the reliability and accuracy of the quadratic model.

**Bioactive and proximate composition of the mycelia**

Table 4 listed the content of the bioactive and proximate composition of the mycelia cultivated in the optimized and NR media. The crude protein content of the mycelia grown in the optimized medium was 38.71%, lower than that (51.82%) of the mycelia cultured in the NR medium. The fiber content in the mycelia was high, reaching 10.51%, 77.97% higher than that of the mycelia (5.93%) grown in the NR medium. The crude ash content of the mycelia grown in the optimized medium was high, reaching 10.43%, while it was only 5.75% in the NR medium-cultured mycelia. Total carbohydrate content (39.03%) of the mycelia cultured in the optimized medium was higher than that (35.29%) of mycelia in the NR medium. The difference in the crude fat content in the mycelia between the two media was little.

The triterpenoids and polysaccharides are the two most important bioactive constituents in *G*. *lucidum.* As shown in Table 4, the mycelia cultivated in the optimal medium had triterpenoid content of 32.44 mg/g, whereas it was only 20.35 mg/g for the mycelia grown in the NR medium. The intracellular polysaccharide content of the mycelia cultured in the optimal medium was high reaching 3.42%, and it was only 2.03% in the mycelia grown in the NR medium. Clearly, there were significant differences in the content of triterpenoids and polysaccharides in the mycelia grown in the optimized and NR media.

**Table 4. Contents of bioactive and proximate composition in the mycelia of *G*. *lucidum* grown** in the optimized medium and the nutrient-rich medium.

| Components | Medium | |
| --- | --- | --- |
|  | Optimized medium | Nutrient-rich medium |
| Moisture (%) | 5.62 ± 0.04a | 5.78 ± 0.04a |
| Crude ash (%) | 10.43 ± 0.33a | 5.75 ± 0.19b |
| Crude fat (%) | 1.32 ± 0.03a | 1.21 ± 0.02b |
| Crude protein (%) | 38.71 ± 0.51b | 51.82 ± 0.64a |
| Crude fiber (%) | 10.51 ± 0.06a | 5.93 ± 0.04b |
| Carbohydrate (%) | 39.03 ± 1.02a | 35.29 ± 0.84b |
| Intracellular polysaccharides (%) | 3.42 ± 0.21a | 2.03 ± 0.11b |
| Triterpenoids (mg/g) | 32.44 ± 2.17a | 20.35 ± 1.56b |

**Content of amino acid compositions in the mycelia**

The content of amino acid compositions of the mycelia cultivated in the optimized medium and the NR medium are listed in Table 5. It can be seen that the total amino acid content in the mycelia cultivated in the optimized medium was 262.4 mg/100g, and the essential amino acid content was 115.4 mg/100 g, while the mycelia grown in the NR medium had much higher content values, with the total amino acid content of 337.1 mg/100 g and the essential amino acid content of 139.5 mg/100 g. The content of all examined amino acid compositions of the mycelia cultivated in the optimized medium were significantly lower than those of the mycelia grown in the NR medium. Among all examined amino acids of the mycelia grown in the two media, the greatest difference in the content value was observed for lysine.

**Table 5. Content of amino acid compositions in the mycelia of *G*. *lucidum* cul**tivated in the optimized medium and the nutrient-rich medium.

| Amino acids | Content (mg/100 g) |  |
| --- | --- | --- |
|  | Optimized medium | NR medium |
| Asparagine | 24.8 ± 0.32b | 32.0 ± 0.78a |
| Threonine* | 13.9 ± 0.21b | 16.6 ± 0.28a |
| Serine | 14.6 ± 0.55b | 19.0 ± 0.42a |
| Glutamate | 37.1 ± 1.25b | 51.6 ± 1.61a |
| Glycine | 15.1 ± 0.34b | 22.1 ± 0.41a |
| Alanine | 18.9 ± 0.27b | 23.2 ± 0.33a |
| Valine* | 23.0 ±0.36b | 25.3 ± 0.20a |
| Methionine* | 6.9 ± 0.12b | 7.5 ± 0.21a |
| Isoleucine* | 12.4 ± 0.16b | 14.3 ± 0.15a |
| Leucine* | 21.7 ± 0.22b | 25.3 ± 0.31a |
| Tyrosine* | 7.7 ± 0.14b | 8.1 ± 0.20a |
| Phenylalanine* | 12.8 ± 0.32b | 15.1 ± 0.18a |
| Lysine* | 17.0 ± 0.24b | 27.3 ± 0.34a |
| Histidine | 6.5 ± 0.07b | 8.1 ± 0.16a |
| Arginine | 16.8 ± 0.21b | 25.0 ± 0.36a |
| Proline | 13.2 ± 0.14b | 16.6 ± 0.17a |
| Total essential acids | 115.4 ± 2.35b | 139.5 ±1.69a |
| Total amino acids | 262.4 ± 3.18b | 337.1 ± 3.26a |

* represents essential amino acids.

Data in each row followed by the different letters are significantly different (*P*>0.05).

**Discussion**

During the past several decades, extensive investigation has been carried out for the production of *G*. *lucidum* mycelia, triterpenoids (or ganoderic acids), and polysaccharides by submerged fermentation technique. However, most of these studies are performed in flasks and laboratory-scale bioreactors, and commercial scale fermentation has not yet been developed successfully*.* The lack of information on submerged cultivation of mushrooms in bioreactors is suggested to be responsible for the limitation of its commercial application [17]. Besides this obstacle, the low productivity may be another major limiting factor for the application of submerged fermentation on a commercial scale. The effective utilization of mycelia and culture broth may be a route to improve the economy of *G*. *lucidum* submerged fermentation process, and thus its industrial scale application will be feasible.

In submerged fermentation of *G*. *lucidum,* the medium compositions used varied significantly in different studies with different purposes. For medium preparation, a variety of biochemical reagents like yeast extract and several inorganic salts such as KH_2_PO_4_ are often utilized [13, 16], and in some cases some low-cost agricultural products such as corn flour and agricultural by-products like soymeal have also been utilized [21], mainly focusing on achieving high yields of biomass and biologically active substances, whereas the safety of the resulting mycelia and broth is often not taken into consideration. Therefore, when these products are used in the food and healthy industries, it may give rise to food safety concerns.

In recent years, the utilization of natural fermentative media has attracted attention in submerged fermentation of *G*. *lucidum.* Sugarcane juice was employed in *G*. *lucidum* submerged fermentation, and found that the concentration of proteins and the percentage of total essential amino acids in the fermentation broth increased, and the broth was developed into a beverage having features of high antioxidant activity and non-toxicity [22]. In a study done by Yang and Zhang [23], soymilk fermented by *G.* *ludicum* exhibited improved acceptability and health properties. A liquid medium comprising of black soybean and *Astragalus membranaceu* was utilized in *G*. *lucidum* fermentation and the generated fermentation broth showed high antioxidant activity [24]. To better utilize resources, cheese whey was used in *G*. *lucidum* fermentation for mycelial production [25]. The pumpkin juice was used to as culture medium, and its nutritional quality was greatly improved [26]. In the present study, three low-lost agricultural products, i.e., potato, sucrose, and corn flour, were utilized as medium ingredients to prepare a food-grade medium by growing *G*. *lucidum* mycelia through submerged fermentation.

Several studies have shown that the composition of culture media had a significant effect on the biosynthesis of bioactive substances of the mycelia during submerged fermentation of medicinal mushrooms like *Antrodia cinnamomea* and *Ganoderma sinense* [27, 28]. In the present study, to provide a scientific basis for the utilization of mycelia cultivated in the natural medium, its nutritional and bioactive composition was investigated. The mycelia contained high contents of triterpenoids and intracellular polysaccharides, significantly higher than that of the mycelia grown in the nutrient-rich medium. Hence, the cultured food-grade mycelia can be used as a desirable material for the extraction of triterpenoids or ganoderic acids and intracellular polysaccharides, which have great potential for use in drug development. Also, the mycelia could be directly processed into capsules as healthy foods.

Dietary fiber is a non-starch polysaccharide that is resistant to digestion and adsorption in the human small intestine, but which can be fermented in the colon. A wide variety of physiological benefits to human body have been reported, such as blood sugar and cholesterol attenuation and antitumor activity [29]. Mushroom is a good source of dietary fiber, and has attracted much attention [30, 31]. Dietary polysaccharides of *G*. *lucidum* had remarkable positive effects on the growth, immune function, and disease resistance in aquatic animals. In the present study, the crude fiber content of the mycelia cultivated in the optimized medium reached 10.51%, significantly higher than that (5.93%) of the mycelia grown in the nutrient-rich medium and also higher than those of other studies [33]. The high content of crude fiber implies that the mycelia obtained from the developed natural medium could be used as a fiber supplement in diet.

In submerged fermentation of mushrooms, the exopolysaccharides (EPS) are often the major target product. As the EPS concentration in broth is generally low, and moreover its separation requires a large amount of organic solvent, the recovery of this biomacromolecule is uneconomic. For the above reason, the direct utilization of fermentation broth may be appropriate. In a recent study done by Bai et al. [34], the direct utilization of an antimicrobial fermentation broth was reported in the postharvest storage of wax apple and guava fruits. Zhao et al. [35] reported that the incorporation of the mycelial culture filtrate could improve the quality Chinese steamed bread. The fermentation broth of *G*. *lucidum* as a novel antioxidant and antimicrobial agent has been reported [36, 37]. In the present study, as all used medium ingredients are natural, the direct use of broth is safe to human body, and there are no concerns regarding food safety problem. Therefore, the fermentation broth obtained in this study is suitable for use as materials in foods.

In the present study, the mycelia cultivated in the natural medium contained a high content of triterpenoids, and thus it may be suitable for use in some specific applications such as for triterpenoids-based drug development. At the same time, the high fiber content of mycelia makes it suitable as a supplement for preparing fiber-rich food or healthy products. The fermentation broth in high volume may be an ideal ingredient for making functional beverages and used as an antimicrobial agent. Therefore, using the natural medium for *G*. *lucidum* submerged fermentation, a zero-waste bioprocess could be established, and its production on the commercial scale would be feasible from a viewpoint of economic benefits.

**Author Contributions**

**Conceptualization:** Shizhong Zheng, Shengrong Liu.

**Formal analysis:** Weirui Zhang.

**Funding acquisition:** Shengrong Liu.

**Investigation:** Shizhong Zheng, Lu Zhang, Yihui Yang.

**Methodology:** Qianhui Huang, Qi Wei.

**Project administration:** Shengrong Liu.

**Supervision:** Shizhong Zheng, Sheng-Rong Liu.

**Writing – original draft:** Shizhong Zheng, Lu Zhang.

**Writing – review & editing:** Shizhong Zheng, Shengrong Liu.

**References**

1. Jong SC, Birmingham JM. Medicinal benefits of the mushroom *Ganoderma*. Adv Appl Microbiol. 1992; 37: 101–134.

2. Hsu KD, Cheng KC. From nutraceutical to clinical trial: frontiers in *Ganoderma* development. Appl Microbiol Biotechnol. 2018; 102: 9037–9051.

3. Mizuno T, Wang GY, Zhang J, Kawagishi H, Nishitoba T, Ti JX. Reishi, *Ganoderma lucidum* and *Ganoderma tsugae*: bioactive substances and medicinal effects. Food Rev Int. 1995; 11: 151–166.

4. Paterson RRM. *Ganoderma*–A therapeutic fungal biofactory. Phytochemistry. 2006; 67: 1985–2001.

5. Zhou XW, Su KQ, Zhang YM. Applied modern biotechnology for cultivation of *Ganoderma* and development of their products. Appl Microbiol Biotechnol. 2012; 93: 941–963.

6. Feng J, Feng N, Yang Y, Liu F, Zhang J, Jia W, et al. Simple and reproducible two-stage agitation speed control strategy for enhanced triterpene production by lingzhi or reishi medicinal mushrooms, *Ganoderma lucidum* ACCC G0119 (higher Basidiomycetes) based on submerged liquid fermentation. Int J Med Mushrooms. 2015; 17: 1151–1159.

7. Feng J, Feng N, Zhang JS, Yang Y, Jia W, Lin CC. A new temperature control shifting strategy for enhanced triterpene production by *Ganoderma lucidum* G0119 based submerged liquid fermentation. Appl Biochem Biotechnol. 2016; 180: 740–752.

8. Yang FC, Liau CB. The influence of environmental conditions on polysaccharide formation by *Ganoderma lucidum* in submerged cultures. Process Biochem. 1998; 33: 547–553.

9. Chang MY, Tsai GJ, Houng JY. Optimization of medium composition for the submerged culture of *Ganoderma lucidum* by Taguchi array design and steepest ascent method. Enzyme Microb Technol. 2006; 38: 407–414.

10. Feng J, Feng N, Tang QJ, Liu YF, Yang Y, Liu F, et al. Optimization of *Ganoderma lucidum* polysaccharides fermentation process for large-scale production. Appl Biochem Biotechnol. 2019; 189: 972–986.

11. Wang XL, Yang HL, Liu GQ. Enhanced triterpene acid production by *Ganoderma lucidum* using a feeding stimulus integrated with a two-stage pH-control strategy. J Chem Technol Biotechnol. 2016; 91: 2974–2982.

12. Feng J, Feng N, Tang QJ, Liu YF, Tang CH, Zhou S, et al. Development and optimization of the triterpenoid and sterol production process with Lingzhi or Reishi medicinal mushroom, *Ganoderma lucidum* strain G0017 (Agaricomycetes), in liquid submerged at large scale. Int J Med Mushrooms. 2021; 23: 43–53

13. Liu GQ, Zhang KC. Enhancement of polysaccharides in *Ganoderma lucidum* by the addition of ethyl acetate extracts from *Eupolyphaga sinensis* and *Catharsius molossus*. Appl Microbiol Biotechnol. 2007; 74: 572–577.

14. Xu JW, Xu YN, Zhong JJ. Production of individual ganoderic acids and expression of biosynthetic genes in liquid static and shaking cultures of *Ganoderma lucidum*. Appl Microbiol Biotechnol. 2010; 85: 941–948.

15. Xu JW, Ji SL, Li HJ, Zhou JS, Duan YQ, Dang LZ, et al. Increased polysaccharide production and biosynthetic gene expressions in a submerged culture of *Ganoderma lucidum* by the overexpression of the homologous α-phosphoglucomutase gene. Bioprocess Biosyst Eng. 2015; 38: 399–405.

16. Liu SR, Zhang WR. Optimization of submerged culture conditions involving a developed fine powder solid seed for exopolysaccharide production by the medicinal mushroom *Ganoderma lucidum*. Food Sci Biotechnol. 2019; 28: 1135–1145.

17. Berovic M, Podgornik BB. Engineering aspects of Lingzhi or Reishi medicinal mushroom *Ganoderma lucidum* (Agaricomycetes) biomass submerged cultivation in bioreactor: a review. Int J Med Mushrooms. 2019; 21: 253–265.

18. Ouzouni PK, Petridis D, Koller WD, Kyriakos A, Riganakos KA. Nutritional value and metal content of wild edible mushrooms collected from West Macedonia and Epirus, Greece. Food Chem. 2009; 115: 1575–1580.

19. Dubois M, Gilles KA, Hamilton JK, Rebers PA, Smith F. Colorimetric method for determination of sugars and related substances. Ana Chem.1956; 28: 350–356.

20. Cai CS, Ma JX, Han CR, Jin Y, Zhao GZ, He XW. Extraction and antioxidant activity of total triterpenoids in the mycelium of a medicinal fungus, *Sanghuangporus sanghuang*. Sci Rep. 2019; 9: 7418.

21. Xu P, Ding ZY, Qian Z, Zhao CX, Zhang KC. Improved production of mycelial biomass and ganoderic acid by submerged culture of *Ganoderma lucidum* SB97 using complex media. Enzyme Microb Tech, 2008; 42: 325–331.

22. Wang Q, Huang Q, Zhang L, Wang L, Hu B, Xu R, et al. Evaluation of a sugarcane juice beverage fermented by *Ganoderma lucidum*: nutritional and antioxidant activity. Food Sci Technol (Campinas). 2022; 42: e84822.

23. Yang H, Zhang L. Changes in some components of soymilk during fermentation with the basidiomycete *Ganoderma lucidum*. Food Chem. 2009; 112: 1–5.

24. Chien YL, Ho CT, Chiang BH, Hwang LS. Effect of fermentation time on antioxidative activities of *Ganoderma lucidum* broth using leguminous plants as part of the liquid fermentation medium. Food Chem. 2011; 126: 1586–1592.

25. Lee H, Song M, Yu Y, Hwang S. Production of *Ganoderma lucidum* mycelium using cheese whey as an alternative substrate: response surface analysis and biokinetics. Biochem Eng J. 2003; 15: 93–99.

26. Zhao J, Liu W, Chen D, Zhou C, Song Y, Zhang Y, et al. Microbiological and physicochemical analysis of pumpkin juice fermentation by the basidiomycetous fungus *Ganoderma lucidum*. J Food Sci. 2015; 80: 241–251.

27. Shih IL, Pan K, Hsieh C. Influence of nutritional components and oxygen supply on the mycelial growth and bioactive metabolites production in submerged culture of *Antrodia cinnamomea*. Process Chem. 2006; 41: 1129–1135.

28. Liu GQ, Ren GP, Wang XL, Zhao Y. Statistical optimization of the key medium components by response surface methodology to promote ganoderic acid formation by medicinal mushroom *Ganoderma sinense* in submerged culture. J Med Plants Res. 2011; 5: 425–431.

29. Cheung PCK. Mini-review on edible mushrooms as source of dietary fiber: preparation and health benefits. Food Sci Hum Well. 2013; 2: 162–166.

30. Peter C, Cheung K. Dietary fiber content and composition of some cultivated edible mushroom fruiting bodies and mycelia. J Agric Food Chem. 1996; 44: 468–471.

31. Zhang Y, Zhu J, Zou Y, Ye Z, Guo L, Zheng Q. Insoluble dietary fiber from five commercially cultivated edible mushrooms: structural, physiochemical and functional properties. Food Bioscience. 2024; 57: 103514.

32. Mohan K, Muralisankar T, Uthayakumar V, Chandirasekar R, Rajan DK. Dietary *Ganoderma lucidum* polysaccharides to enhance the growth, immune response and disease resistance of freshwater prawn *Macrobrachium rosenbergii*. Aquacult Rep. 2019; 14: 100203.

33. Zhou QL, Yang W, Lin JF, Guo LQ. Optimization of medium pH, growth media compositions and analysis of nutritional components of *Ganodema lucidum* in submerged culture fermentation. Eur J Med Plants. 2015; 6: 17–25.

34. Bai JL, Wang HH, Zhang JM, Wu QP, Mo SP, He YL, et al. Postharvest quality maintenance of wax apple and guava fruits by use of a fermented broth of an ε-poly-L-lysine-producing *Streptomyces* strain. PLoS ONE. 2022; 17: e0265457.

35. Zhao G, Wei L, Liu Y, Wang H. Impact of the fermentation broth of *Ganoderma lucidum* on the quality of Chinese steamed bread. AMB Expr. 2019; 9: 133.

36. Ćilerdžić J, Kosanić M, Stajić M, Vukojević J, Ranković B. Species of genus *Ganoderma* (Agaricomycetes) fermentation broth: a novel antioxidant and antimicrobial agent. Int J Med Mushrooms. 2016; 18: 397–404.

37. Mohan K, Muralisankar T, Uthayakumar V, Chandirasekar R, Karthick Rajan D. Sarnthima R, et al. Culture broth of *Ganoderm lucidum* exhibited antioxidant, antibacterial and α-amylase inhibitory activities. J Food Sci Technol. 2017; 54: 3724–3730.
