# Supplementary material for: Optimization of a natural fermentative medium for submerged mycelial culture of Ganoderma lucidum and the nutritional and bioactive composition of the cultured food-grade mycelia
Source: PLoS One. 2026 Jan 29;21(1):e0337539. doi: 10.1371/journal.pone.0337539 (PMC12854441; doi:10.1371/journal.pone.0337539)
Supplement: Attachment — Submitted filename: PONE-D-25-34815 Responses to the reviewer.doc [file pone.0337539.s005.doc]

**Responses to the reviewer’s comments**

Manuscript number: PONE-D-25-34815

**Title:** Optimization of a natural fermentative medium for submerged mycelial culture of *Ganoderma lucidum* and the nutritional and bioactive composition of the cultured food-grade mycelia

**Dear Editors,**

We appreciate your consideration and giving us the opportunity to revise our manuscript. The manuscript has been revised according to the comments and suggestions, and all problems required have been addressed. Revised portion are marked in red in the paper. Point-by-point responses to these comments are showed below in this letter.

Sincerely yours,

Shizhong Zheng, Lu Zhang, Yihui Yang, Shengrong Liu, Qi Wei, Qianhui Huang, Weirui Zhang

**Editors: Some douts, suggestions and recommendation**

**Comment 1:** The Manuscript “Optimization of a natural fermentative medium for submerged mycelial culture of *Ganoderma lucidum* and the nutritional and bioactive composition of the cultured food-grade mycelia” is a nice study. However, the manuscript requires some corrections suggested in the review track mode by the reviewrs, and also follow the grammers and language editing for better shaping the manuscript.

**Response:** Thanks. We have carefully checked the manuscript.

**Reviewer 1#: Some douts, suggestions and recommendations**

**Comment 1:** **Line 127-128** “Why corn flour was using. Is there any scientific evidence for using the corn flour”.

**Response:** Accept. corn flour is widely used in the fermentation industry, and for culturing mushroom strains. We have cited several references to support our choice for use of corn flour. See in the revision.

**Comment 2: Line 181:** “Have you seen the proximate analysis for the same under basic media”.

**Response:** Your suggestion is greatly appreciated. As the difference in the medium nutrition level especially nitrogen source between the optimized medium and the basic medium was not large, we selected a nitrogen-rich medium as a reference for comparison. The proximate composition analysis of the cultured mycelia between the two media was carried out, and there were significant differences in chemical compositions of the mycelia between the two media.

**Comment 3: Line 182:** “What is the added advantage of your supplementations”.

**Response:** Thanks. In mushroom submerged liquid fermentation, the mycelial biomass is an important index. In the present research, the addition of corn flour was adopted mainly for promoting mycelial growth.

**Reviewer 2#: Some douts, suggestions and recommendations**

**Comment 1: Line 22:** “What is the need of this research? Explain it is to develop for what?”

**Response:** Accept. The objective of the present study has been added in the revised manuscript.

**Comment 2: Line 66-68:** This has to be reflect in abstract session.

**Response:** Thanks. This part is cited from the reference, it was not appropriate to remove to the abstract session.

**Comment 3: Line 303:** “Usually, PDA or PDB will be the basic media for mushroom fungal culture. Have you check for PDA to compare the optimized one?”.

**Response:** Thanks. PDB is often used as a basic medium for mushroom fungal culture. In the present research, after the medium optimization, the biomass yield was greatly enhanced as compared with PDB medium. On the other hand, the difference in the medium nutrition level especially nitrogen source between the optimized medium and PDB was not large, we therefore selected a nitrogen-rich medium as a reference for comparison.

**List of the changes in revision**

**Abstract**

**1. Line 21-22 of page 2,** “with healthy-promoting benefits and pharmacological properties” was changed as “with health-promoting and pharmacological properties”.

**2. Line 22-23 of page 2,** “To safely utilize the mycelia and broth of *G*. *lucidum* by submerged fermentation” was changed as “The present study aimed to provide a liquid medium in *G*. *lucidum* submerged fermentation suitable for safely utilizing the mycelia and broth and as a solution to potentially improve the process’ economy.”.

**3. Line 23-25 of page 2,** “a natural fermentative medium was formulated and optimized by the one-variable-at-a-time approach and response surface methodology (RSM)” was changed as “For this, a natural medium was formulated and optimized.”.

**4. Line 26-27 of page 2,** “were compared” was changed as “were also determined”.

**5. Line 27 of page 2,** “ tested” was inserted .

**6. Line 28 of page 2,** “additional” was deleted.

**7. Line 29-31 of page 2,** “A further optimization by RSM indicated that the optimal medium was comprised of 300 g/L potato (use its extract), 25 g/L sucrose, and 7.5 g/L corn flour” was changed as “A further study by response surface methodology obtained an optimal medium comprising of 300 g/L potato (use its extract), 25 g/L sucrose, and 7.5 g/L corn flour”.

**8. Line 31 of page 2,** “With” was changed as “using”.

**9. Line 32-34 of page 2,** “The crude ash (10.43%) and fiber (10.51%) content in the mycelia cultivated with the optimal medium was much higher than that (5.75% and 5.93%) of the mycelia grown in the NR medium” was changed as “The crude ash (10.43%) and fiber (10.51%) content of the mycelia cultivated in the optimal medium was higher than those (ash 5.75% and fiber 5.93%) of the mycelia grown in the NR medium”.

**10. Line 35 of page 2,** “the latter” was changed as “the mycelia cultivated in the NR medium”.

**11. Line 36-38 of page 2,** “The mycelia grown the optimized medium had 32.24 mg/g triterpenoids and 3.42% intracellular polysaccharides, significantly higher than that (20.35 mg/g and 2.03%) of the mycelia by growing in the NR medium” was changed as “The mycelia had the content of triterpenoids at 32.24 mg/g and intracellular polysaccharides at 3.42%, higher than those (triterpenoids 20.35 mg/g and intracellular polysaccharides 2.03%) of the mycelia cultivated with the NR medium”.

**12. Line 38-40 of 2,** “These findings proved that the food-grade cultured mycelia had the potential for use in the pharmacological industry because of its high content of triterpenoids and polysaccharides” was changed as “These findings indicated that the cultured food-grade mycelia had the potential for use in the pharmacological industry”.

**13. Line 40-41 of page 2,** “and as a dietary supplement in the food and healthy products” was deleted.

**14. Line 41 of page 2,** “Meanwhile, the broth had the potential for safe use in foods due to free of chemicals” was inserted.

**15. Line 41-44 of page 2,** “The present study provides a reference for improving the economic benefits of *G*. *lucidum* submerged culture by using a natural medium, providing the condition for the safe utilization of all fermentation products, and thus its large-scale industrial fermentation may be economically feasible” was changed as “The present study provides a reference for improving the economy of *G*. *lucidum* submerged culture by using a natural medium, and its industrial fermentation may be economically viable”.

**16. Line 44 of page 2,** “**Keywords:** Lingzhi, medium optimization, mycelium, triterpenoids, polysaccharides” was inserted.

**Introduction**

**1. Line 47 of page 3,** “famous” was inserted.

**2. Line 55 of page 3,** “compounds” was deleted.

**3. Line 56 of page 3,** “ in this fungus” was inserted.

**4. Line 57 of page 3,** “To meet the high demand for high-quality *G*. *lucidum* in market” was changed as “To meet the great demand for high-quality *G*. *lucidum* products in market”.

**5. Line 58 of page 3,** “solid” was inserted.

**6. Line 58 of page 3,** “is a widely adopted method” was changed as “widely adopted”.

**7. Line 59 of page 3,** “With the fruiting bodies as raw materials” was changed as “With the fruiting bodies and spores as raw materials”.

**8. Line 59 of page 3,** “wide” was added.

**9. Line 60-61 of page 3,** “could be manufactured” was changed as “have been developed and manufactured ”.

**10. Line 61 of page 3,** “solid” was inserted.

**11. Line 62 of page 3,** “some” was changed as “several”.

**12. Line 62-63 of page 3,** “there are several disadvantages associated with solid cultivation” was changed as “it should be pointed out that the solid cultivation of *G*. *lucidum* has several disadvantages”.

**13. Line 68 of page 3,** “in the form of mycelia and bioactive metabolites” was inserted.

**14. Line 68-70 of page 3-4,** “In submerged fermentation of mushrooms, the mycelia and small quantities of several useful metabolites could be obtained” deleted.

**15. Line 70 of page 4,** “healthy-promoting” was changed as “health-promoting”.

**16. Line 71-72 of page 4,** “considerable attention has been paid to its submerged cultures for the production of mycelia, polysaccharides and triterpenoids” was changed as “its submerged culture has received considerable attention, mainly for the production of mycelia, polysaccharides and triterpenoids”.

**17. Line 75 of page 4,** “development of solid seed” was changed as “utilization of solid seed”.

**18. Line 76 of page 4,** “some improvements” was revised as “significant improvements”.

**19. Line 76 of page 4**, “However” was deleted.

**20. Line 77-79 of page 4,** “the productivity of mycelia and bioactive metabolites are still low in submerged fermentation of *G*. *lucidum*, making the process industrially unattractive” was revised as “the productivity of both mycelia and bioactive metabolites are still low, making the submerged fermentation process of *G*. *lucidum* industrially unattractive”.

**21. Line 79-82 of page 4,** “Increasing product yields and developing novel production systems by solving the existing problems associated with submerged fermentation technique of mushrooms are considered to be crucial for its success on a commercial scale” was changed as “Another obstacle is that there are many technical and engineering problems associated with *G*. *lucidum* submerged fermentation in bioreactors”.

**22. Line 82 of page 4,** “In submerged fermentation of mushrooms” was deleted.

**23. Line 82-84 of page 4,** “utilizing all the fermentation products including mycelia and culture broth may be another solution to improve bioprocess economy” was changed as “Under these circumstances, the effective utilization of all the fermentation products including mycelia and culture broth may be a reasonable solution to improve the bioprocess’ economy”.

**24. Line 84-85 of page 4,** “but the effect of medium compositions on the safety of fermentation products has not yet been considered when they were selected” was deleted.

**25. Line 86-88 of page 4,** “to develop a natural medium for submerged mycelial culture of *G*. *lucidum* from a perspective for the safe utilization of fermentation products (mycelia and culture broth)” was revised as “to provide a desirable medium for submerged mycelial culture of *G*. *lucidum* suitable for the safe utilization of its fermentation products (mycelia and culture broth)” .

**26. Line 89 of page 4,** “then” was inserted.

**27. Line 91 of page 4,** “Furthermore” was inserted.

**28. Line 91 of page 4,** “better” was added.

**29. Line 92 of page 4,** “harvested from the developed natural medium” was deleted.

**30. Line 93 of page 4,** “in the mycelia” was deleted.

**31. Line 93-96 of page 4-5,** “The present study provides a new perspective for improving the economic benefits of *G*. *lucidum* submerged fermentation by using a natural medium, ultimately leading to the safe utilization of all fermentation products” was revised as “The present study provides a new perspective to perform *G*. *lucidum* submerged fermentation by using a natural medium for the safe utilization of all the fermentation products, thus could potentially improve the economy of process”.

**32. Line 96 of page 5,** “and thus its large-scale industrial fermentation may be feasible” was revised as “making its large-scale industrial fermentation economically feasible”.

**Materials and methods**

**1. Line 98 of page 5,** “and medium” was deleted.

**2. Line 100 of page 5,** “used in this study” was changed as “throughout this study”.

**3. Line 105 of page 5,** “For comparison”was inserted”.

**4. Line 106 of page 5,** “Nutrient-rich (NR) medium was comprised of ” was changed as “a nutrient-rich (NR) medium was used, which was comprised of”.

**5. Line 109 of page 5,** “actively” was inserted.

**6. Line 112 of page 5,** “mycelial” was deleted.

**7. Line 116-117 of page 5,** “in subsequent studies” was deleted.

**8. Line 120 of page 6,** “The inoculation of liquid seed was set at 10% (v/v)” was changed as “ The inoculation level of liquid seed was all set at 10% (v/v)”.

**9. Line 121-122 of page 6,** “The samples were taken for harvesting mycelia” was changed as “The samples were taken for estimating mycelial biomass”.

**10. Line 123 of page 6,** “One-variable-at-one-time experiments” was changed as “One-variable-at-one-time experimental design”.

**11. Line 124 of page 6,** “in *G*. *lucidum* submerged cultures” was inserted.

**12. Line 124-125 of page 6,** “To select a suitable carbon source for mycelial yield of *G*. *lucidum* in submerged cultures” was changed as “To select a suitable carbon source for mycelial growth”.

**13. Line 125 of page 6,** “the glucose in the medium” was changed as “the glucose in the basic medium”.

**14. Line 126 of page 6,** “At the end of submerged cultivation, the biomass production was measured, and then a desirable carbon source was selected” was inserted.

**15. Line 126-127 of page 6,** “After this” was changed as “After the screening”.

**16. Line 127 of page 6,** “easily available” was inserted.

**17. Line 128 of page 6,** “with the optimal carbon source” was changed as “with the selected carbon source”.

**18. Line 129 of page 6,** “based on biomass yield” was inserted.

**19. Line 141 of page 6,** “in submerged cultures” was inserted.

**20. Line 141-142 of page 6-7,** “Corn powder” was changed as “Corn flour”.

**21. Line 142 of page 7,** “2” was changed as “2.5”.

**22. Line 143 of page 7,** “The matrix of BBD design” was revised as “The matrix of BBD”.

**23. Line 145 of page 7,** “The submerged culture experiments were performed as previously described” was inserted.

**24. Line 153 of page 7,** “The statistical significance of the equation” was revised as “The significance of the obtained regression model”.

**25. Line 158 of page 7,** “indicates” was revised as “indicate”.

**26. Line 159 of page 7,** “were statistically significant” was changed as “were considered statistically significant”.

**27. Line 162 of page 7,** “the type of interactions between the variables” was changed as “the type of interaction between the two variables”.

**28. Line 169 of page 8,** “For production of mycelia” was changed as “For the production of mycelia”.

**29. Line 169 of page 8,** “in flasks” was changed as “in 250 mL flasks”.

**30. Line 170 of page 8,** “Submerged cultivation was also performed using the NR medium” was changed as “For comparison, the NR medium was also tested in the fermentation”.

**31. Line 171-172 of page 8,** “The mycelial cultures were filtered for harvesting mycelia, and washed with distilled water” was changed as “The mycelial cultures were filtered with gauze and completely washed with distilled water”.

**32. Line 173 of page 8,** “were used for analysis” was changed as “ were used for the chemical analysis”.

**33. Line 175-178 of page 8,** “three independent culture flasks” was changed as “three independent flask cultures”.

**34. Line 178 of page 8,** “The dry mycelial weight” was changed as “The weight of dry mycelia”.

**35. Line 187-188 of page 9,** “by subtracting the contents of ash, fat, and protein from 100” was changed as “ by subtracting the percentage contents of ash, fat, and protein from 100%”.

**36. Line 188-189 of page 9,** “Amino acid analysis was carried out after hydrolysis of the mycelia with 6 mol/L HCl for 24 h at 110°C using an amino acid analyzer” was changed as “ Amino acid analysis was carried out using an amino acid analyzer after hydrolysis of the mycelia with 6 mol/L HCl for 24 h at 110°C”.

**37. Line 199-200 of page 9,** “The powder (100 mg) was extracted twice in 5 mL 80% ethanol in one week” was changed as “The powder (100 mg) was extracted twice in one week, each in 5 mL 80% ethanol”.

**38. Line 201 of page 9,** “for analysis” was revised as “in the reaction”.

**39. Line 205-206 of page 9,** “by Duncan’s multiple range tests (*P* < 0.05)” was revised as “by Duncan’s multiple range tests and Student’s *t*-test at *P* < 0.05”.

**Results**

**1. Line 212 of page 10,** “Among the tested carbon sources,” was changed as “Among various tested carbon sources”.

**2. Line 213 of page 10,** “and maltose, glucose and fructose supported moderate mycelial growth” was revised as “followed by maltose, glucose and fructose with moderate growth”.

**3. Line 213-214 of page 10,** “and the least growth of only 1.68 g/L was observed with starch” was revised as “and the least growth of only 1.68 g/L was shown when starch was used”.

**4. Line 215 of page 10,** “Considering the biomass yield, material cost as well as availability” was revised as “Considering the cost and availability of the carbon sources as well as the biomass yield, sucrose was therefore selected as a favorable carbon source”.

**5. Line 217 of page 10,** “The results are in agreement with the findings of previous studies in which sucrose was shown to be an excellent carbon source in submerged fermentation of *G*. *lucidum* for production of biomass and bioactive substances” was inserted.

**6. Line 218 of page 10,** “the” was deleted.

**7. Line 219 of page 10,** “The different letters” was changed as “Different letters”.

**8. Line 225 of page 10,** “Fig 2 shows that” was revised as “As shown in Fig 2”.

**9. Line 225 of page 10,** “additional” was deleted.

**10. Line 227 of page 10,** “reached ” was revised as “attained”.

**11. Line 229 of page 10,** “obtained in the medium (3.44 g/L) without addition” was revised as “obtained in the control medium (without addition)”.

**12. Line 231 of page 10,** “In the literature, corn flour as a carbon source is frequently used in mushroom submerged fermentation, and shown to be effective in promoting mycelial growth and triterpenoids biosynthesis” was inserted.

**13.** **Line 231 of page 10,** “Their findings are generally consistent with our results” was inserted.

**14. Line 235 of page 11,** “of biomass yield” was deleted.

**15. Line 236-237 of page 11,** “in BBD design for further optimization by RSM” was revised as “in BBD experiments for further optimization by RSM”.

**16.** **Line 237-238 of page 11,** “The design matrix of BBD along with the corresponding response values of biomass yield were shown in Table 2” was changed as “The matrix of BBD along with the corresponding response values (biomass yield) are shown in Table 2”.

**17. Line 240 of page 11,** “shown” was inserted.

**18. Line 240 of page 11,** “The difference” was changed as “The great difference”.

**19. Line 242 of page 11,** “in submerged culture” was changed as “in submerged cultures”.

**20. Line 244 of page 11,** “experimental” was deleted.

**21. Line 247 of page 11,** “quadratic” was deleted”

**22. Line 252 of page 12,** “is” was changed as “was”.

**23. Line 262 of page 12,** “was low” was changed as “was not high”.

**24. Line 272-273 of page 13,** “as the slope of the curvature was relatively flat” was changed as “as the slope of the curvature was low”.

**25. Line 282-283 of page 13,** “The curvature of response surfaces in Fig 3C is relatively flat, and its corresponding contour plots are circular in shape (Fig 3c)” was deleted.

**26. Line 285-286 of page 13,** “Changing sucrose concentration within the investigated range had little effect on biomass yield” was changed as “The curvature of response surface between sucrose (*X_2_*) and corn flour (*X_3_*) in Fig 3C indicated that changing sucrose concentration within the investigated region had little effect on biomass yield”.

**27. Line 286-287 of page 13**, “whereas a small variation of corn flour concentration could lead to a remarkable impact on the response variable, with the preference at higher concentration” was changed as “Contrary to this, a small variation in corn flour amount would lead to a considerable impact on the response variable (biomass yield), with the preference at higher concentration”.

**28. Line 289 of page 14,** “The contour plots between sucrose and corn flour was circular in shape (Fig 3c), implying that there were no significant interactions between these two variables” was inserted.

**29. Line 290-293 of page 14,** “With the numerical optimization technique of the software Design-Expert and based on response surface analysis, the optimal concentration of the three compositions for maximal biomass yield was attained as 300 g/L potato, 25 g/L sucrose, and 7.5 g/L corn flour” was changed as “ Using the numerical optimization technique provided by the software Design-Expert and based on response surface analysis, the optimal levels of the three investigated compositions for maximal biomass yield were attained as 300 g/L potato, 25 g/L sucrose, and 7.5 g/L corn flour” and moved to Line 298 of page 14.

**30. Line 302 of page 14,** “Medium composition had a significant effect on the content of nutritional and bioactive compositions in the mycelia of several mushroom strains during submerged fermentation” was inserted,

**31. Line 302 of page 14,** “Table 4 listed” was revised as “Table 4 lists”

**32. Line 308-310 of page 14,** “Total carbohydrate content (39.03%) of the mycelia cultured in the optimized medium was higher than that (35.29%) of mycelia in the NR medium” was changed as “Total carbohydrate content of the mycelia cultured in the optimized medium was 39.03%, higher than that (35.29%) of mycelia harvested from the NR medium”.

**33. Line 313-315 of page 15,** “the mycelia cultivated in the optimal medium had triterpenoid content of 32.44 mg/g, whereas it was only 20.35 mg/g for the mycelia grown in the NR medium” was revised as “the mycelia cultivated in the optimal medium had a triterpenoid content of 32.44 mg/g, while it was only 20.35 mg/g in the mycelia in the case of grown in the NR medium”.

**34. Line 315-317 of page 15,** “The intracellular polysaccharide content of the mycelia cultured in the optimal medium was high reaching 3.42%, and it was only 2.03% in the mycelia grown in the NR medium” was revised as “The intracellular polysaccharide content was high, reaching 3.42%, and 2.03% in the mycelia grown in the NR medium”.

**35. Line 317-319 of page 15,** “there were significant differences in the content of triterpenoids and polysaccharides in the mycelia grown in the optimized and NR media” was changed as “there existed significant differences in the content of triterpenoids and polysaccharides between the mycelia grown in the optimized and NR media”.

**36. Line 321 of page 15,** “the nutrient-rich medium” was changed as “the NR medium”

**37. Line 322 of page 15,** “cultured” was inserted.

**38. Line 324 of page 15,** “It can be seen that” was deleted.

**39. Line 327-328 of page 15,** “with the total amino acid content of 337.1 mg/100 g and the essential amino acid content of 139.5 mg/100 g” was revised as “with the total amino acid content being 337.1 mg/100 g and the essential amino acid content being 139.5 mg/100 g”.

**40. Line 331-332 of page 15,** “the greatest difference in the content value was observed for lysine” was revised as “ the greatest difference in the content was shown by lysine, arginine and glutamate in the mycelia between the two media”.

**41. Line 336 of page 15,** “the” was deleted.

**Discussion**

**1. Line 338-348 of page 16-17,** All of the content from Line 338 to 348” was deleted.

**2. Line 349 of page 17,** “It is well known that medium is a crucial factor for the growth of microbes and the production of useful metabolites during microbial fermentation” was inserted.

**3. Line 349-350 of page 17,** “In submerged fermentation of *G*. *lucidum,* the medium compositions used varied significantly in different studies with different purposes” was changed as “As for submerged fermentation of *G*. *lucidum,* a large number of medium have been used, and the compositions used among them varied significantly”.

**4. Line 350 of page 17,** “Presently” was inserted.

**5. Line 350-352 of page 17,** “For medium preparation, a variety of biochemical reagents like yeast extract and several inorganic salts such as KH_2_PO_4_ are often utilized” was changed as “a variety of biochemical reagents like yeast extract and peptone, and several common inorganic salts such as KH_2_PO_4_ and MgSO_4_ are often utilized in medium development for *G*. *lucidum* fermentation”.

**6. Line 352 of page 17,** “in some cases” was deleted.

**7. Line 355-356 of page 17,** “whereas the safety of the resulting mycelia and broth is often not taken into consideration” was revised as “while the safety of the mycelia and broth resulting from the bioprocess has often not been taken into consideration”.

**8. Line 356-357 of page 17,** “Therefore, when these products are used in the food and healthy industries, it may give rise to food safety concerns” was changed as “If these fermentation products are directly used in food and health products, it may give rise to some concerns of food safety”.

**9. Line 358-359 of page 17,** “In recent years, the utilization of natural fermentative media has attracted attention in submerged fermentation of *G*. *lucidum*” was revised as “Currently, there are a few number of available reports regarding the use of natural fermentative media in submerged fermentation of *G*. *lucidum*”.

**10. Line 366 of page 17,** “generated” was deleted.

**11. Line 367-368 of page 17,** “cheese whey was used in *G*. *lucidum* fermentation for mycelial production” was changed as “cheese whey was used as medium for fermentative production of *G*. *lucidum* mycelia”.

**12. Line 368 of page 17,** “The pumpkin juice was used to as culture mediumt” was changed as “The pumpkin juice was used as a culture medium in *G*. *lucidum* fermentation”.

**13. Line 371-372 of page 17-18,** “by growing *G*. *lucidum* mycelia through submerged fermentation” was revised as “for growing *G*. *lucidum* mycelia, and showed excellent performance”.

**14. Line 373 of page 18,** “Several studies have shown that” was revised as “It has been reported that”.

**15. Line 375 of page 18,** “medicinal” was deleted.

**16. Line 376-377 of page 18,** “to provide a scientific basis for the utilization of mycelia cultivated in the natural medium” was revised as “in order to provide a scientific basis for the better utilization of mycelia cultivated in the optimized natural medium”.

**17. Line 381 of page 18,** “the extraction of triterpenoids or ganoderic acids and intracellular polysaccharides” was revised as “the extraction of triterpenoids (or ganoderic acids) and polysaccharides”.

**18. Line 382 of page 18,** “Also” was revised as “Besides this usage”.

**19. Line 382-383 of page 18,** “the mycelia could be directly processed into capsules as healthy foods” was revised as “the mycelia had the potential to be directly processed into capsules as health products because of its high content of bioactive substances”.

**20. Line 385-387 of page 18,** “A wide variety of physiological benefits to human body have been reported, such as blood sugar and cholesterol attenuation and antitumor activity” was revised as “A wide variety of physiological benefits such as blood sugar and cholesterol attenuation and antitumor activity to human body by dietary fiber have been reported”.

**21. Line 392-393 of page 18,** “significantly higher than that (5.93%) of the mycelia grown in the nutrient-rich medium and also higher than those of other studies” was revised as “significantly higher than that of other studies”.

**22. Line 393-395 of page 18,** “The high content of crude fiber implies that the mycelia obtained from the developed natural medium could be used as a fiber supplement in diet” was revised as “The high content of crude fiber implies that the obtained mycelia could be used as a fiber supplement in diet”.

**23. Line 396-397 of page 18-19,** “In submerged fermentation of mushrooms, the exopolysaccharides (EPS) are often the major target product” was changed as “The exopolysaccharides (EPS) are often the major target product in submerged fermentation of mushrooms”.

**24. Line 397 of page 19,** “As the EPS concentration in broth is generally low” was revised as “however, the EPS concentration in broth is generally low”.

**25. Line 398 of page 19,** “moreover its separation requires a large amount of organic solvent” was revised as “Furthermore, the separation of EPS requires a large amount of organic solvent”.

**26. Line 398-399 of page 19,** “the recovery of this biomacromolecule is uneconomic” was revised as “Considering these facts, the recovery of this biomacromolecule is uneconomic”.

**27. Line 399-400 of page 19,** “For the above reason, the direct utilization of fermentation broth may be appropriate” was revised as “As an alternative, the direct utilization of fermentation broth has been attempted”.

**28. Line 400-402 of page 19,** “In a recent study done by Bai et al. [34], the direct utilization of an antimicrobial fermentation broth was reported in the postharvest storage of wax apple and guava fruits” was deleted.

**29. Line 404 of page 19,** “and also as an enzyme inhibition agent” was inserted.

**30. Line 405-407 of page 19,** “as all used medium ingredients are natural, the direct use of broth is safe to human body, and there are no concerns regarding food safety problem” was revised as “as the medium ingredients used are all natural, the broth obtained from *G*. *lucidum* fermentation on the natural medium had characteristics of high safety to human body”.

**31. Line 407-408 of page 19,** “Therefore, the fermentation broth obtained in this study is suitable for use as materials in foods” was revised as “Therefore, the fermentation broth holds great promise for use as a low-cost material in various fields such as the food industry”.

**32. Line 409-414 of page 19,** The content from Line 409 to 414 was deleted.

**33. Line 414 of page 19,** “In conclusion, a natural fermentative medium was developed and optimized for performing *G*. *lucidum* submerged fermentation” was inserted.

**34. Line 414 of page 19,** “The mycelia obtained on the natural medium after *G*. *lucidum* fermentation contained high content of bioactive substances, holding the great promise for use in the pharmacological and health industries” was inserted.

**35. Line 414 of page 19,** “Meanwhile, the fermentation broth had the great potential for safe use in various fields such as the food industry” was inserted.

**36. Line 414-417 of page 19,** “Therefore, using the natural medium for *G*. *lucidum* submerged fermentation, a zero-waste bioprocess could be established, and its production on the commercial scale would be feasible from a viewpoint of economic benefits” was changed as “By using the natural medium in *G*. *lucidum* submerged fermentation, a zero-waste bioprocess could be well established, and its industrial production may be economically feasible”.

**Supporting information**

**1. Line between 417 and 418 of page 19, “**Supporting information” was inserted.

**2. Line 418,** “S1 Raw data for figure illustration” was inserted.

**3. Line 418,** “(XLSX)” was inserted.

**References**

**1. Line 489 of page 22,** reference “21. Wei ZH, Liu LL, Guo XF, Li YJ, Hou BC, Fan QL, et al. Sucrose fed-batch strategy enhanced biomass, polysaccharide, and ganoderic acids production in fermentation of *Ganoderma lucidum* 5.26. Bioprocess Biosyst Eng. 2016; 39: 37–44” was inserted.

**2. Line 492of page 22,** reference “Wu XJ, Hansen C. Effects of whey permeate-based medium on the proximate composition of *Lentinus edodes* in the submerged culture. J Food Sci. 2010; 71: 174–179” was inserted.

**3. Line 492 of page 22,** reference “ Zhu ZY , Liu XC, Tang YL, Dong FY, Sun HQ, Chen L, et al. Effects of cultural medium on the formation and antitumor activity of polysaccharides by *Cordyceps gunnii*. J Biosci Bioeng. 2016; 122: 494–498” was added.

**4. Line 527-528 of page 24,** reference “34. Bai JL, Wang HH, Zhang JM, Wu QP, Mo SP, He YL, et al. Postharvest quality maintenance of wax apple and guava fruits by use of a fermented broth of an ε-poly-L-lysine-producing *Streptomyces* strain. PLoS ONE. 2022; 17: e0265457” was deleted.
